# Putative Roles of Plant-Derived Tannins in Neurodegenerative and Neuropsychiatry Disorders: An Updated Review

**DOI:** 10.3390/molecules24122213

**Published:** 2019-06-13

**Authors:** Ghulam Hussain, Jia Huang, Azhar Rasul, Haseeb Anwar, Ali Imran, Javeria Maqbool, Aroona Razzaq, Nimra Aziz, Ehtisham ul Haq Makhdoom, Muhsin Konuk, Tao Sun

**Affiliations:** 1 Neurochemicalbiology and Genetics Laboratory (NGL), Department of Physiology, Faculty of Life Sciences, Government College University, Faisalabad 38000, Pakistan; drhaseebanwar@gcuf.edu.pk (H.A.); javeria_maqbool@yahoo.com (J.M.); aroonarazzaq@yahoo.com (A.R.); nimra.aziz1794@gmail.com (N.A.); shaamisaahib@gmail.com (E.u.H.M.); 2 Center for Precision Medicine, School of Medicine and School of Biomedical Sciences, Huaqiao University, Xiamen 361021, China; huangjia5970@163.com; 3 Department of Zoology, Faculty of Life Sciences, Government College University, Faisalabad 38000, Pakistan; drazharrasul@gmail.com; 4 Institute of Home and Food Sciences, Government College University, Faisalabad 38000, Pakistan; aliimran.ft@gmail.com; 5 Faculty of Engineering and Natural Sciences, Department of Molecular Biology and Genetics, Uskudar University, Istanbul 34662, Turkey; muhsin.konuk@uskudar.edu.tr

**Keywords:** neurodegenerative diseases (NDDs), neuropsychiatric diseases, Alzheimer’s disease (AD), Parkinson’s disease (PD), depression

## Abstract

Neurodegenerative and neuropsychiatric diseases are characterized by the structural and functional abnormalities of neurons in certain regions of the brain. These abnormalities, which can result in progressive neuronal degeneration and functional disability, are incurable to date. Although comprehensive efforts have been made to figure out effective therapies against these diseases, partial success has been achieved and complete functional recovery is still not a reality. At present, plants and plant-derived compounds are getting more attention because of a plethora of pharmacological properties, and they are proving to be a better and safer target as therapeutic interventions. This review aims to highlight the roles of tannins, ‘the polyphenol phytochemicals’, in tackling neurodegenerative diseases including Alzheimer’s and Parkinson’s diseases as well as neuropsychiatric disorders like depression. Among the multifarious pharmacological properties of tannins, anti-oxidative, anti-inflammatory, and anti-cholinesterase activities are emphasized more in terms of neuroprotection. The current review also throws light on mechanistic pathways by which various classes of tannins execute neuroprotective effects. Despite their beneficial properties, some harmful effects of tannins have also been elaborated.

## 1. Introduction

Phytochemicals are naturally occurring bio-active compounds which are produced by plants. They are involved in modulating metabolic pathways and are not only significant for conferring various health-promoting effects but also for being effective against several diseases including neurodegenerative and neuropsychiatric disorders [1,2]. They exhibit various pharmacological properties, such as anti-oxidant, anti-aging, anti-cancer, anti-hyperglycemic, and anti-bacterial behaviors, and many others [3,4]. Among these activities, anti-inflammatory and anti-oxidative properties are more prominent. Moreover, these compounds can ameliorate the pathogenesis of various neurodegenerative diseases (NDDs) such as Alzheimer’s disease (AD) and Parkinson’s disease (PD) [2,5]. The major classes of phytochemicals include alkaloids, flavonoids, terpenes, and tannins. Here, we will discuss only tannins in detail.

Tannins, which are polyphenol compounds, are a diversified group of phytochemicals with many health-related beneficial effects [6]. They are mostly found in vegetables and fruits and are therefore consumed vastly on a daily basis with different consumption rates from region to region. Reported ingestion of tannins in Indian regions ranges from 1.5–2.5 g/day [7] and about 1 g/day in the USA [8]. They have a molar mass of up to 20000 D and their chemistry varies depending on their source. Their distribution and concentration are highly different in the different parts of a plant, such as in the leaves, roots, seeds, and fruits. Tannins are a white or light-yellow amorphous powder and exhibit an astringent taste with a strange smell. Tannins have attracted the attention of the scientific community for their beneficial effects owing to their anti-oxidative capacity. This is a highly valuable property as it can reduce oxidative damage that is a major hallmark of almost every disease.

## 2. Health Benefits of Tannins

Tannins are biologically potent compounds in relation to the regulation of health-associated aspects of all living beings, including humans. They can be effective in the treatment of various diseases by virtue of the multitude of their beneficial activities [9]. These properties include anti-inflammatory [10], anti-oxidative [11], anti-convulsant [12], and antitumor [13] behaviors, as well as many others. Importantly, they have been shown to be effective in the pathogenesis of various NDDs such as AD, PD, and multiple sclerosis (MS) [14,15,16]. In normal physiological processes and during cell metabolism, reactive oxygen species (ROS) and free radicals are produced which, under certain conditions, can cause various diseases like cancer, diabetes, cardiovascular diseases, stroke, PD, and AD [17]. Both endogenous and exogenous antioxidants try to maintain redox homeostasis by detoxification and scavenging of ROS or by blocking ROS production [18,19]. They can accelerate blood clotting, reduce blood pressure, decrease serum lipid levels, and inhibit the activity of gastrointestinal nematodes [20]. They are protective against cardiovascular diseases by inhibiting platelet aggregation [21]. They exhibit the potential to cure non-insulin dependent diabetes mellitus by inhibiting adipogenesis and increasing glucose uptake [22]. Moreover, their anti-carcinogenic capability in the digestive tract and inner organs has also been reported. They have also been used for the treatment of diarrhea, dysentery, hemorrhoids, and throat infections [23].

**Figure 1:**
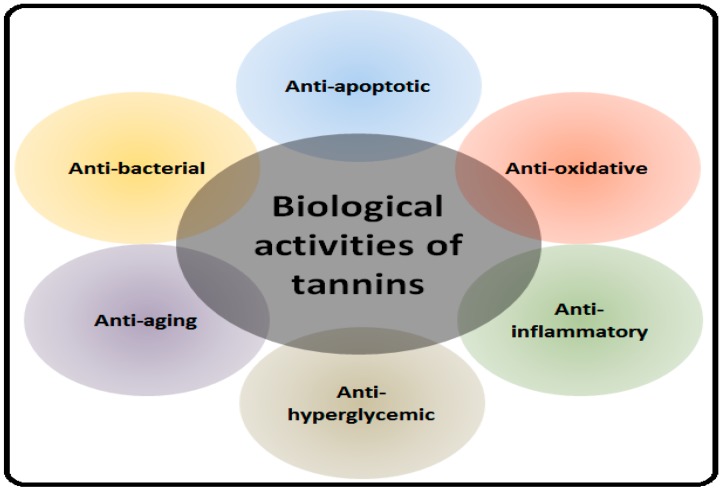
Biological activities of tannins.

Natural products endure a promising source of tannins with biochemical specificity and diverse molecular characteristics which make them appropriate for the accent of multiple signaling pathways in the plethora of different pathological conditions including NDDs [24,25]. The protective role of tannins in NDDs is described in the following sections along with possible underlying mechanisms. The health-promoting effects of tannins are highlighted in Figure 1.

## 3. Classification of Tannins

Tannins are categorized into various classes depending upon their chemical structure and nature of the functional group. Chemical and physical properties of these classes of tannins are also different. They are soluble in glycerol, alcohol, acetone, and water. They are dark brown, brown, and/or reddish in color with a puckering and bitter taste, and are non-crystalline. Chemically, tannins can react with salts and are considered strong anti-oxidants. Due to their structural diversity, a systemic classification of tannins is done on the basis of chemical properties and specific structural characteristics [26].

**Figure 2:**
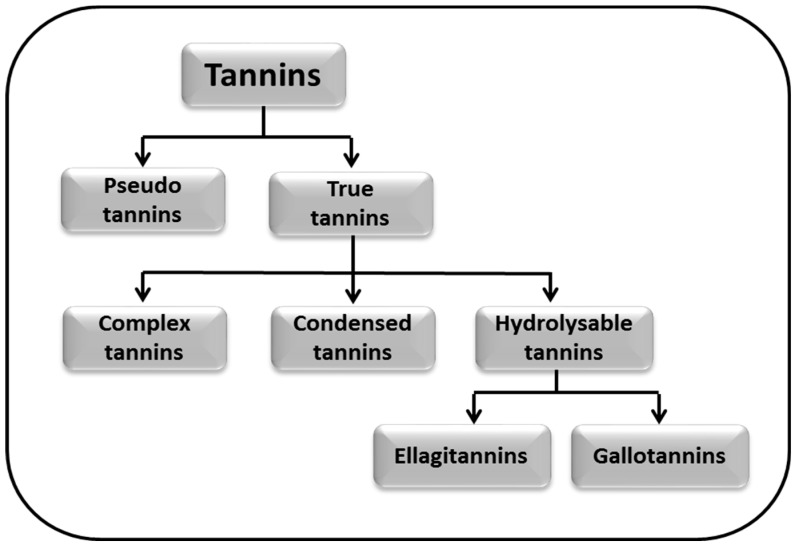
Classification of tannins.

Tannins are classified into two major classes: pseudo tannins and true tannins. True tannins are complex phenolic compounds and may be further sub-classified into complex tannins, condensed tannins (CTs), and hydrolysable tannins (HTs). Hydrolysable tannins are the major polyphenols generally found in pomegranate juice which account for about 90% of its antioxidant activity; hydrolysable tannins are further classified into ellagitannins (ETs) and gallotannins [27]. Moreover, the classification of tannins is given in the Figure 2.

Different types of tannins are present in edible plants, fruits, and vegetables. Depending upon their occurrence ratio in these sources, their consumption rate is highly diversified, as discussed above. The classification and sources of different tannin groups are given in Table 1.

## 4. Neurodegenerative Diseases

**Figure 3:**
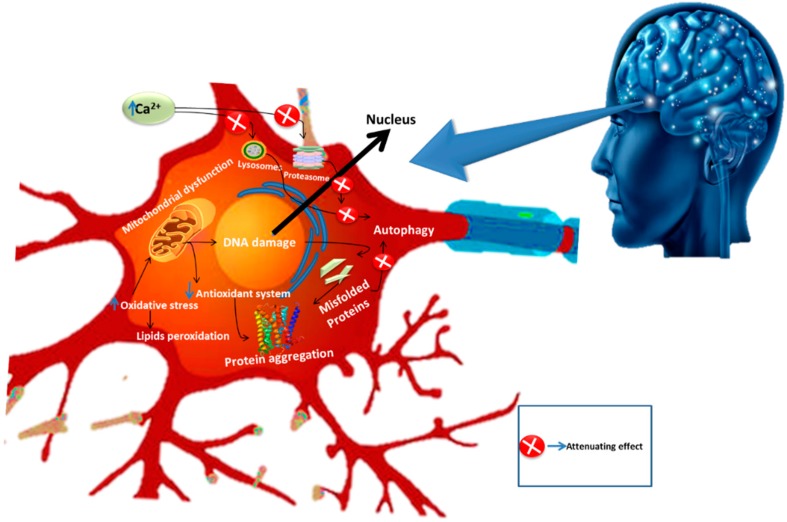
Pathological pathways of neurodegenerative diseases.

Neurodegeneration is the progressive loss of neuronal cells in certain regions of the brain. Brain ailments fall amongst the chief clinical problems both in developed and developing countries [41]. The majority of NDDs share some common characteristics such as incurable damage to various cell types including astrocytes and microglial activity [2,42]. In these diseases, proteins with altered physicochemical properties are accumulated in the brain. This phenomenon not only affects the neurons but also the glial cells [42,43]. The common pathological pathways involved in NDDs are somewhat similar or dissimilar but eventually cause the altered functioning of neuroglia cells and ultimately neuronal demise. While investigating the pathophysiology of these diseases, neuroscientists have explored numerous underlying pathways such as impaired calcium homeostasis, abnormal functioning of astrocytes, oxidative stress, and neuro-inflammation. All of these underlying pathways collectively lead to neuronal cell death when altered and this neurodegeneration is defined under the umbrella of NDDs. Despite extensive research towards understanding and developing novel approaches against NDDs, modern advancement in therapeutic intervention is still far behind in providing an effective treatment for these ailments. Interestingly, phytochemicals have successfully drawn the attention of researchers, who have explored their positive aspects against these diseases [43]. The major contributors in the pathogenesis of NDDs are illustrated in Figure 3.

### 4.1. Alzheimer’s Disease and Its Pathogenesis

Alzheimer’s disease, a chronic degenerative disease of the brain, is the most common cause of dementia. It is a syndrome with a wide range of symptoms including difficulties of language, memory, problem-solving, and various other cognitive skills. In the pathogenesis of AD, neuronal cells or synapses in the cerebral cortex and certain subcortical regions are destroyed [44]. Moreover, pathogenesis involves the progressive attenuation of memory, learning, and cognitive functions [45] caused by the aggregation of amyloid-β (Aβ) peptide, tau protein hyperphosphorylation, and eventually oxidative stress [46]. During disease progression the affected areas exhibit deposition of senile plaques mainly composed of Aβ peptides. Additionally, gamma-secretase-mediated sequential cleavages of the amyloid precursor protein (APP) and β-secretase (beta-site amyloid precursor protein cleaving enzyme (BACE)) also generate Aβ40/42 [47,48,49]. In most NDDs, aggregated proteins are considered the prime factors involved in neuronal cell death. Two types of protein aggregation appear in AD: insoluble extracellular aggregates mainly formed by hyperphosphorylation of tau protein (tau) and insoluble extracellular aggregates resulting from Aβ peptides. Thus, all of these factors flare up the pathological process of AD, ultimately resulting in reduced synaptic transmission and exaggerated neuronal loss. Furthermore, the pathophysiology of NDDs coincides with alterations at the cellular level, followed by the dysfunctional voltage-gated ion channels. On these grounds, it can be stated that the brain’s electrical activity alters significantly because of these factors in AD [50,51,52]. Despite the high prevalence of this disease, available treatments are still unable to significantly obstruct the progression of disease and offer only a small window of symptomatic benefit [53].

### 4.2. Parkinson’s Disease and Its Pathogenesis

**Figure 4:**
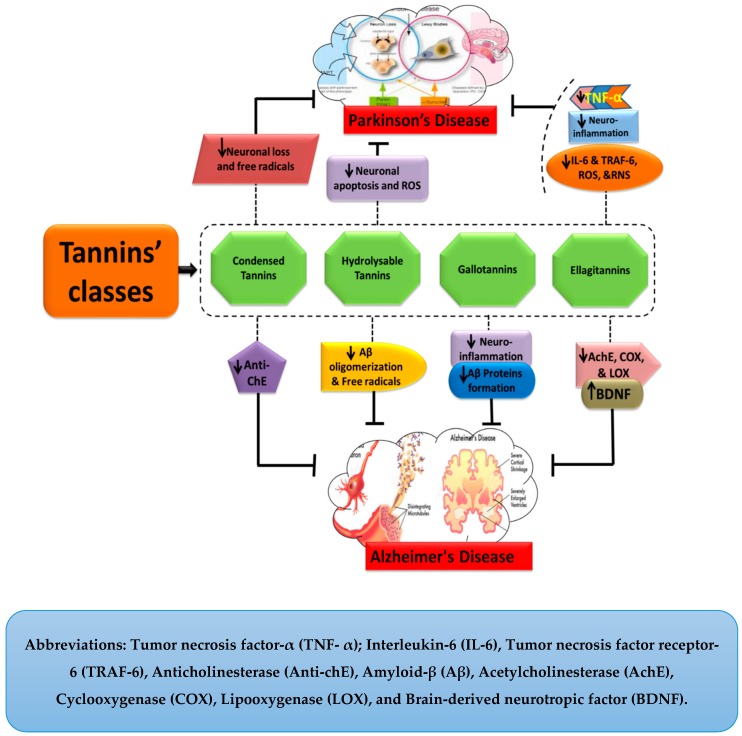
Neuroprotective effects of tannins.

Parkinson’s disease is a sporadic progressive NDD with an unestablished etiology. It is manifested by functional deficits of dopamine in the substantia nigra (SN). Amongst several causes, head trauma, inflammation, oxidative stress, accumulation of free radicals and exposure to environmental toxins are taken as the important mediators of this disease. It has been stated that cognitive impairment and depression also occur, followed by the degeneration of neural cells [54,55]. Symptoms of PD may include resting tremors, rigidity, slowness of movement (bradykinesia) with difficulty in walking, postural instability, and behavioral problems [56]. Microglial activation and neuro-inflammation are also associated with the pathogenesis of PD. Oxidative stress induced by nitric oxide (NO), ROS, and decreased mitochondrial activity are also considered major players that are essentially involved in the pathogenesis of PD [57]. Microglial activation leads to the formation of cytotoxic factors including NO, tumor necrosis factor-α (TNF-α), and interleukin-1β (IL-1β), and hence can cause neurodegeneration [58]. Some of the major underlying mechanisms involved in neuronal cell death and associated NDDs are shown in Figure 4.

### 4.3. Depression and Its Pathogenesis

Depression is a mood disorder with a multitude of signs, symptoms, and causes. It is one of the most prevalent diseases affecting the population. It is a life-threatening and disabling illness which recruits several pathological pathways that may lead to neurodegeneration. Although mood disorders are not typical neurodegenerative diseases, studies have shown that reduced neurogenesis and neuroplasticity may occur [59]. The general symptoms of depression include weight loss, disturbance of sleep cycles, irritability, anger, self-loathing, and loss of energy. There are many types of depression depending upon the causes, symptoms, and underlying molecular pathways. In a subject suffering from major depressive disorder (MDD), abnormalities in the GABAergic amino-acid neurotransmitter (AANt) and glutamatergic neurotransmission system have been observed. Similarly, the role of glial cells in regulating both of these neurotransmission systems seems chiefly relevant to the pathological processes of mood disorders. More precisely, the compromised functions of glial cells are speculated to upregulate glutamatergic activation, particularly at extrasynaptic spaces which have been revealed to play a neurotoxic role [60]. Moreover, neuro-inflammation also contributes to the pathogenesis of depression on a very broad scale. The key factor underlying depression and neuro-inflammation lies within the dysregulation of production and discharge of both pro-inflammatory and anti-inflammatory cytokines. This can come from sources either external or internal to the concerned system [61]. Oxidative stress is another potent mediator of depression via multiple aspects. Increased ROS and reactive nitrogen species (RNS) resulting from elevated levels of oxidative stress cause severe damage to the nucleotides of deoxyribonucleic acid and alter production of neurotransmitters. They also cause oxidation of glycine and thereby flare up the progression of depression [62].

## 5. Tannins and Their Neuroprotective Functions

Tannins are biological molecules with numerous pharmacological properties, including, in particular neuroprotection. They ameliorate the symptoms of AD and PD by retarding their pathogenesis through the attenuated production of various cytotoxic factors. As in case of AD, gallotannins hinder the accumulation of Aβ peptides and thereby offer a protective role against the disease’s pathogenesis and progression. Figure 2 illustrates the general mechanistic approaches of tannins against AD and PD. Moreover, a detailed description of their mechanism has been discussed in the following sections.

### 5.1. Pseudo Tannins

Pseudo tannins, which are low molecular weight compounds, are naturally found in catechu, tea, coffee, and *Strychnos Nux. Vomica* (*S. Nux. Vomica*). These compounds are different from CTs and HTs due to their chemical characteristics. For example, they do not change their color while undergoing a goldbeater’s skin test as the other previously mentioned classes do [63]. Neuroprotective properties of pseudo tannins are a prominent point of discussion. The protective role of pseudo tannins in AD is described below.

#### Pseudo Tannins in Alzheimer’s Disease

It is a well-established fact that the pathological pathways of AD are very complex and their regulation in multiple dimensions has made them difficult to take over, due to which AD remains incurable till date. This class of tannins exhibits a protective role in AD. One of the chief sources of pseudo tannins is *S. Nux. Vomica* and this is highly effective against AD. In this aspect, it can check the rate of progression by up-regulating the acetylcholine (Ach) binding to its receptors and thereby stimulating the neurotransmission [28]. There is a great dearth of data regarding the neuroprotective effects of pseudo tannins and further research is crucially needed to elaborate on the role of pseudo tannins against NDDs, and especially AD.

### 5.2. True Tannins

This group of tannins is divided into different subclasses depending upon the structural and functional qualities that are described below.

#### 5.2.1. Complex Tannins

Complex tannins are a subclass of true tannins with flavone as the basic unit. They are predominantly found in legumes, nuts, corn, rice, and tea. They are formed by the combination of an ellagitannin or gallotannin unit and catechin [27]. Regarding their health promoting benefits, including mainly their neuroprotective effects, there is a scarcity of data and extensive research is suggested.

#### 5.2.2. Condensed Tannins

Condensed tannins (CTs) are polymers that are produced by the condensation of flavans. They are also called proanthocyanidins. They are the polymers of flavan-3-ol and are eminent naturally occurring antioxidants. Condensed tannins are polyphenols in nature and are found in fruits, seeds, nuts, vegetables, bark, flowers, red wine, and black tea [64]. These compounds are described as strong anti-oxidants as they can scavenge free radicals and ameliorate damage associated to free radicals’ associated [65]. Their effective role in attenuating the pathogenesis of AD and PD is described here.

##### Condensed Tannins in Alzheimer’s disease

The CTs are believed to play pivotal roles in slowing the advancement of NDDs; including AD and PD. Increased oxidative stress is considered a major hallmark underlying these brain ailments. These compounds are taken as crucial factors than can halt the pathogenesis of various NNDs through their multiple domains. Condensed tannins have been found to exhibit anticholinesterase activity and portray their neuroprotective effects in AD [29,30].

##### Condensed Tannins in Parkinson’s disease

**Figure 5:**
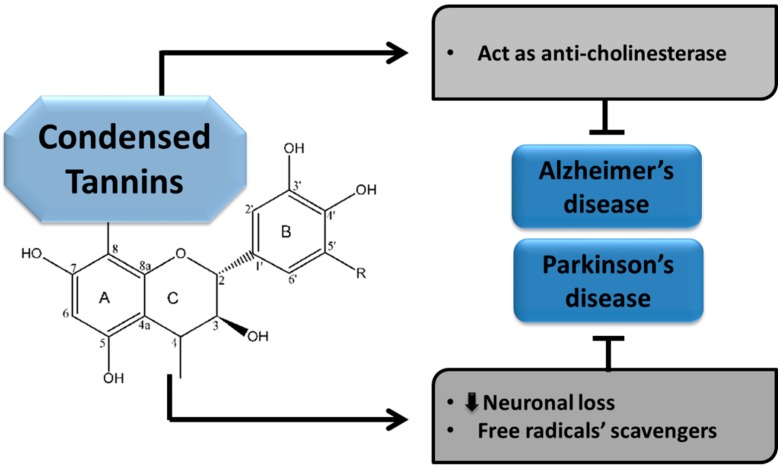
Neuroprotective effects of condensed tannins.

The CTs have also been described as potent remedial agents which arrest the progress of PD in different ways. They exhibit anti-oxidative properties owing to their capability of scavenging free radicals and thus helping to retard the pathogenesis of PD [66]. As CTs are the richest sources of polyphenols, most of their activities are derived from polyphenolic compounds. Among the various plant sources of CTs, one of the rich plant sources, *Myracrodruon urundeuva* (SEMU), exhibits neuroprotective properties in the case of PD. According to an available study, PD pathogenesis was significantly improved with reduced brain lesions in a 6-hydroxydopamine (6-OHDA) induced AD model. Moreover, the number of viable neurons was also increased. The 6-OHDA acts as an inhibitor of mitochondrial respiratory chain complex I and is widely used for inducing a nigrostriatal lesion [31,32]. Although various studies have been conducted to evaluate the neuroprotective roles of CTs, further work is needed to explore their activities and underlying mechanisms. Different mechanisms of CTs involved in the protection of NDDs, such as AD and PD, are presented below in Figure 5.

#### 5.2.3. Hydrolysable Tannins

The HTs are an important class of tannins with polyhydric alcohol as a core part of their structure. They contain gallic acid in their structure and are a type of phenolic acid. There are two further subdivisions of HTs: ellagitannins and gallotannins. The HTs are found in terrestrial plants, bananas, grapes, and other fruits as well [67,68]. Regarding the intended general health properties of HTs, they are described as anti-inflammatory, anti-oxidant, and anti-angiogenic agents. Their health-related properties may be associated with their polyphenolic constituents, such as Gallic acid. The anti-oxidative property of HTs is helpful in tapering the oxidative stress related to neuron damage. Moreover, it also inhibits the free radicals’ production along with oligomerization of Aβ in case of AD and improves cognitive functions. It can be stated that HTs have potent therapeutic effects for AD but further research is required to give detailed insights [69].

With regards to the role of HTs in NDDs, they prevent both memory and dopaminergic neuronal loss in SN and reduce the level of apoptosis in the hippocampus [70]. The alpha-synuclein (α-Syn) heaps are the chief constituent of Lewy bodies and represent the specific pathological features of PD. Interestingly, gallic acid from the HTs is positively associated with the degradation of α-syn in PD [71]. In oxidative stress, the oxidation of dopamine generates ROS and an unbalanced production of ROS induces neuronal damage, finally leading to neuronal cell death. Gallic acid and its derivatives act as potent antioxidants and free radical scavengers. Thus, they help to ameliorate the motor dysfunctions associated with PD by their anti-oxidative capability [72].

##### Ellagitannins

Ellagitannins, a diverse class of HTs, are polyphenols and are diverse in nature. These compounds hydrolyze into ellagic acid (EA) under in vivo physiological conditions. This EA is metabolized gradually by intestinal microbiota. It is present in seeds (pomegranates), nuts, some berries (strawberries, black raspberries, and raspberries), almonds, and walnuts. Both ETs and EA exert countless health-promoting effects. According to some studies, ET- and EA-rich food intakes can protect against some chronic diseases [73,74].

For instance, a plant named *Terminalia chebula* Retz (*T. chebula*), a major source of CTs, ETs, and polyphenols, has been reported for its anticholinesterase, anti-inflammatory, and antioxidant properties against AD. It has been reported that methanolic extract of *T. chebula* exhibits inhibitory effects on AChE which in turn leads to Ach accumulation in the synaptic cleft and thereby helps in accelerating neurotransmission [33]. Similar effects have been reported by other studies and this effect is considered a result of ETs and EA presence in *T. chebula* [35,75]. Moreover, the anti-inflammatory activities of *T. chebula* have also been reported. It works by inhibiting inflammatory enzymes’ activity, such as that of 5-lipoxygenase (5-LOX) and cyclooxygenase (COX). Similar studies support the fact that ethanolic extract of *T. chebula* inhibits both of these enzymes’ activity [33,75]. In this aspect, *T. chebula* has been found to exert strong anti-oxidant activity and free radicals’ scavenging capability. It also protects neuronal loss by maintaining the levels of brain-derived neurotrophic factor (BDNF) and superoxide dismutase (SOD) [33,76]. Based on these facts, one can conclude that ETs and EA from plant sources can serve as a potential treatment strategy for AD and further work for therapeutic benefits is strongly suggested.

**Figure 6:**
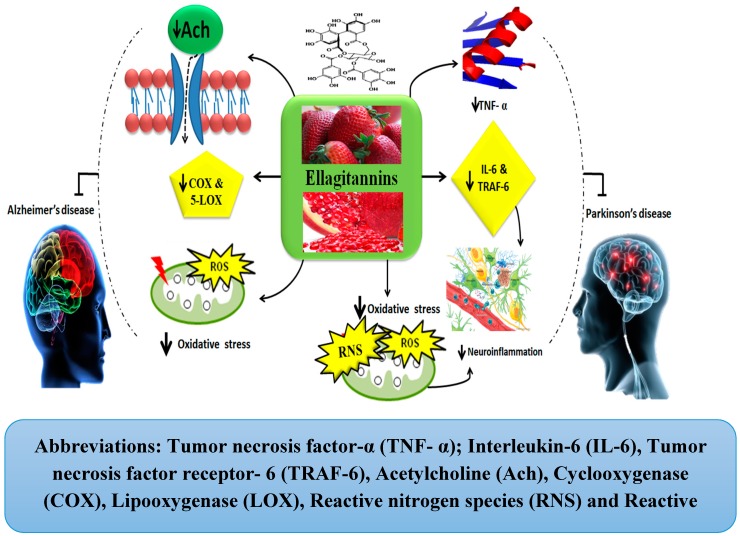
Ellagitannins in Alzheimer’s and Parkinson’s diseases.

The ETs are also involved in providing neuroprotection against PD. The pomegranate, a primary source of ETs, acts as an inhibitor of inflammatory mediators such as tumor necrosis factor receptor 6 (TRAF-6) and other pro-inflammatory cytokines including IL-6 and TNF-α. Thus, it helps to ameliorate the pathogenesis of PD by inhibiting neuro-inflammation [36]. With regard to its anti-oxidative property, it also inhibits the production of free radicals including RNS and ROS [36]. Thus, pomegranates can help in the attenuation of PD pathogenesis. Moreover, berry fruits, another prime source of ETs, also help to improve both cognitive and motor functions in PD. They do so by the modulation of signaling pathways involved in neurotransmission, cell survival, and upregulated neuroplasticity [77]. Similarly, strawberries also inhibit inflammation mediating enzymes, including COX-1 and COX-2 [37,78,79]. Thus, in the light of these facts, it can be concluded that ETs exhibit the ability to overcome the pathological pathways of PD, but future studies are strongly needed to elaborate on their therapeutic effects. The mechanisms involved in the neuroprotection by ETs against AD and PD have been shown in Figure 6.

##### Gallotannins

Gallotannins, another subclass of HTs, consist of a sugar moiety and a gallic acid unit. They are polymers of galloyl units bounded with diverse polyol units [80]. Gallotannins and its derivatives exert neuroprotective effects.

##### Gallotannins in Alzheimer’s disease

Since oxidative damage is a foremost mediator of neuronal death in NDDs, antioxidants are considered the prime factor amongst other therapeutics. Similarly, owing to their antioxidant properties, tannins appear to be a beneficial candidate for neuroprotection. Likewise, gallotannins purified from oak galls exhibit strong anti-oxidative activity leading to neuronal survival. Furthermore, in relation to NDDs such as AD, gallotannins have been found to downregulate Aβ deposition through decreasing β-carboxyl cleavage from the APP, thereby helping to control neuro-inflammation. Interestingly, tannic acids/gallotannins not only restrict the formation of Aβ42 but also destabilize the already formed Aβ42 in AD [38,39,40]. From these factors, the gallotannins group appears to be a potential candidate for neuroprotection in AD. Although a few of the underlying mechanisms have been described, concerned data for concise or discursive pathways leading to neuroprotection is still lacking. Hence, further research is highly needed to sow the seeds for more reliable data for therapeutic interventions.

### 5.3. Tannic Acid in Depression

Tannic acid is a polyphenolic compound and is one of the most important types of the tannins. It acts as a weak acid and is found in the nutgalls formed by insects on twigs of certain oak trees (*Quercus infectoria* and other Quercus species). This compound is described as a strong antidepressant due its activity in reducing neurodegeneration and inhibiting monoamine oxidase [81]. Depression is a common psychiatric disorder affecting a large number of people. It is often difficult to diagnose its causes, making it also difficult to properly and efficiently treat depression. It is a long-lasting illness that disturbs a person’s mood, thoughts, and physical attitude, ultimately leading to a vicious cycle of severe depression [82]. Many plants and formulations have been used to treat depression for thousands of years. Thus, plant-originated compounds open a new window into the treatment of depression. The water extract of the plant named *Terminalia chebula* contains tannins and phenolic contents. This water extract has good antioxidant activities and has the potential to treat depression [83]. This antidepressant property might be attributed to the presence of tannic acid in the extract. Tannic acid has been shown to be a non-selective inhibitor of monoamine oxidase, and, hence, it increases the levels of monoaminergic neurotransmitters in the brain. The polyphenols and tannic acid present in *Terminalia chebula* have an ability to attenuate the oxidative stress during depression.

*Terminalia catappa* L. (TC) belongs to the Combretaceae family and is used as folk medicine in many regions of the world. Its supplementation can suppress stress and depression by regulating monoamine neurotransmitters, BDNF, CREB, AchE, and cortisol levels, as well as by tempering oxidative stress. Hence, TC can be used as a complementary medicine against depression-like disorders [84]. Numerous antidepressants like selective monoamine reuptake inhibitors, serotonin reuptake inhibitors, and monoamine oxidase inhibitors have been used to treat depression. Unfortunately, synthetic antidepressants exhibit adverse effects, and, therefore, have very limited application [85]. With this in mind, the development of herbal-based drugs for the treatment of depression which have comparatively less or no side effects compared to synthetic anti-depressants is necessary [86]. Supplementation of TC successfully reduces monoamine oxidase-A (MAO-A) and monoamine oxidase-B (MAO-B) activities. Thus, TC bestows antidepressant-like effects via inhibition of MAO’s activity in the hippocampus, which then regulates the monoamine neurotransmitter levels [84,87].

## 6. Negative Effects of Tannins on Health

In this review, we have highlighted the neuroprotective roles of tannins in NDDs and have also elaborated on different underlying mechanisms. Although in recent years tannins have begun to reveal their positive aspects to scientists, but compounds have also been reported for their hazardous effects on human health. Overdoses of tannic acids can exert side effects, including nausea, stomach irritation, and liver damage. Similarly, higher consumption of tannins from herbs, such as betel nuts and herbal teas, has been reported to result in an elevated chance of developing throat and oesophageal cancer, but other reports have also demonstrated that the carcinogenic activity of these tannins are due to other components associated with the tannins rather than tannins themselves [88]. Moreover, a high dose of tannins leads to excessive astringency on the mucous membrane, and, thus, it causes irritating effects. This probably involves the underlying mechanism of adding milk into tea, whereby the tannins bind with the milk protein instead of binding with the gut wall. Chronic intake of high levels of tannins is also associated with the onset of constipation [89]. Furthermore, chronic high intake of tannins inhibits the activity of digestive enzymes, i.e., mainly the membrane-bounded enzymes present on the mucosa of the small intestine [6]. In the light of these facts, it may be concluded that tannins can exhibit harmful effects on human health despite their enormous beneficial effects. Further studies are needed to circumvent these reported adverse effects of tannins to get most out of these regarding widely reported beneficial effects, especially for NDDs.

## 7. Conclusions and Future Perspectives

Tannins are water-soluble polyphenols that are present in many plants. They are biologically active compounds that exhibit an influential role in the modulation of physiological aspects of well-being. They not only promote general well-being owing to their health-promoting effects on the living system but at the same time, they inhibit innumerable underlying pathological mechanisms involved in the progression of various diseases. Recent scientific literature supports the conclusions that neurodegenerative diseases are accompanied by oxidative stress, inflammation, aberrant protein accumulation, and mitochondrial dysfunctions. Moreover, these pathological changes lead to disturbed physiological mechanisms which contribute to the etiology of NDDs like PD and AD, and to some extent depression. Therefore, the objective approach behind the term neuroprotection is to restrict the pathological pathways leading to neuronal damage at the molecular level, including neuro-inflammation, oxidative stress, aberrant cellular signaling, mitochondrial dysfunction, and protein aggregation. In this regard, the pivotal neuroprotective effects of tannins are related to their capacity to act as free radical scavengers and to activate the antioxidant system in the body, thereby including protection against neurotoxins and oxidative-stress-induced neuronal damage, neuronal inflammation, and some other factors. Keeping all of these facts in mind regarding neuroprotection, this review has highlighted all of the possible physiological roles of tannins. Despite a huge volume of literature regarding polyphenols, including tannins, their biological effects are still in question due to their dubious bioavailability. The prevention and treatment of NDDs with complex mechanisms need novel targeted therapeutic strategies and future tannin research must target the elaboration of these described benefits at the subcellular and molecular levels to obtain clinical acceptance of all their health-promoting benefits from in vitro, animal model, and preclinical studies. Furthermore, tannins must be probed in depth regarding their risk assessment and safety evaluation in relation to their future pharmacological use in neurodegenerative diseases.

## Figures and Tables

**Table 1:** Classification, sources and neuroprotective roles of tannins.

**Tannins**	**Classes**	**Sub-Classes**		**Mechanism of Action in NDDs**	
				**Alzheimer’s Disease**	**Parkinson’s Disease**
	**Pseudo tannins**	—		Stimulate neurotransmission and upregulate Ach binding with receptors [28].	—
	**True tannins**	Complex tannins		—	—
		Condensed tannins		Exhibit anti-chE activity [29,30].	Anti-oxidants (free radicals scavengers) and attenuate neuronal loss [31,32].
		Hydrolysable tannins	Ellagitannins	Inhibit AChE, COX, and LOX activity, free radicals’ scavengers, maintain BDNF and SOD levels, attenuate neuronal damage [33,34,35].	Attenuate neuro-inflammation, inhibit IL-6, TNF-α, TRAF-6, ROS, and RNS [36]. Upregulate neuroplasticity [37].
			Gallotannins	Attenuate oxidative stress, Aβ formation and deposition, and neuro-inflammation [38,39,40].	—

Abbreviations: Acetylcholine (Ach), Acetylcholinesterase (AchE), Tumor necrosis factor-α (TNF- α); Interleukin-6 (IL-6), Tumor necrosis factor receptor- 6 (TRAF-6), Anticholinesterase (Anti-chE), Amyloid-β (Aβ), Cyclooxygenase (COX), Lipooxygenase (LOX), and Brain-derived neurotropic factor (BDNF), and Superoxide dismutase (SOD).

## References

[1] Hussain G., Rasul A., Anwar H., Aziz N., Razzaq A., Wei W., Ali M., Li J., Li X. (2018). Role of plant derived alkaloids and their mechanism in neurodegenerative disorders. Int. J. Biol. Sci..

[2] Hussain G., Zhang L., Rasul A., Anwar H., Sohail M., Razzaq A., Aziz N., Shabbir A., Ali M., Sun T. (2018). Role of plant-derived flavonoids and their mechanism in attenuation of Alzheimer’s and Parkinson’s diseases: An update of recent data. Molecules.

[3] Aziz N., Rasul A., Malik S.A., Anwar H., Imran A., Razzaq A., Shaukat A., Kashif S., Kamran S., De Aguilar J.G. (2019). Supplementation of *Cannabis sativa* L. leaf powder accelerates functional recovery and ameliorates haemoglobin level following an induced injury to sciatic nerve in mouse model. Pak. J. Pharm. Sci..

[4] Kennedy D.O., Wightman E.L. (2011). Herbal extracts and phytochemicals: Plant secondary metabolites and the enhancement of human brain function. Adv. Nutr..

[5] Uriarte Pueyo I., Calvo M.I. (2009). Phytochemical study and evaluation of antioxidant, neuroprotective and acetylcholinesterase inhibitor activities of *Galeopsis ladanum* L. extracts. Pharmacogn. Mag..

[6] Ky I., Le Floch A., Zeng L., Pechamat L., Jourdes M., Teissedre P.L. (2015). Tannins. Encyclopedia of Food and Health.

[7] Kumari M. (2017). Screening of Potential Sources of Tannin and Its Therapeutic Application. Int. J. Nutr. Food Sci..

[8] Makkar H., Siddhuraju P., Becker K. (2007). Plant Secondary Metabolites: Tannins. Methods Mol. Biol..

[9] Delimont N.M., Haub M.D., Lindshield B.L. (2017). The Impact of Tannin Consumption on Iron Bioavailability and Status: A Narrative Review. Curr. Dev. Nutr..

[10] Thomas M.L.R.M.G., Filho J.M.B. (1985). Anti-inflammatory actions of tannins isolated from the bark of *Anacardwm occidentale* L.. J. Ethnopharmacol..

[11] Wafa N., Sofiane G., Mouhamed K. (2016). The antioxidant and antimicrobial activities of flavonoids and tannins extracted from Phlomis bovei De Noé. Pelagia Res. Libr. Eur. J. Exp. Biol..

[12] Yazdi A., Sardari S., Sayyah M., Ezzati M.H. (2011). Evaluation of the anticonvulsant activity of the leaves of glycyrrhiza glabra var. glandulifera grown in Iran, as a possible renewable source for anticonvulsant compounds. Iran. J. Pharm. Res..

[13] Al-Ayyoubi S., Gali-Muhtasib H. (2007). Anti-tumor signaling pathways modulated by plant polyphenols. New Cell Apoptosis Res..

[14] Basli A., Soulet S., Chaher N., Mérillon J.-M., Chibane M., Monti J.-P., Richard T. (2012). Wine Polyphenols: Potential Agents in Neuroprotection. Oxidative Med. Cell. Longev..

[15] Bora K.S., Arora S., Shri R. (2011). Role of *Ocimum basilicum* L. in prevention of ischemia and reperfusion-induced cerebral damage, and motor dysfunctions in mice brain. J. Ethnopharmacol..

[16] Kashiwada Y., Huang L., Kilkuskie R.E., Bodner A.J., Lee K.-H. (1992). New hexahydroxydiphenoyl derivatives as potent inhibitors of HIV replication in H9 lymphocytes. Bioorganic Med. Chem. Lett..

[17] Winterbourn C.C. (2008). Reconciling the chemistry and biology of reactive oxygen species. Nat. Chem. Biol..

[18] Han X., Shen T., Lou H. (2007). Dietary polyphenols and their biological significance. Int. J. Mol. Sci..

[19] Weiss E.I., Houri-Haddad Y., Greenbaum E., Hochman N., Ofek I., Zakay-Rones Z. (2005). Cranberry juice constituents affect influenza virus adhesion and infectivity. Antivir. Res..

[20] Chung K.-T., Wong T.Y., Wei C.-I., Huang Y.-W., Lin Y. (1998). Tannins and Human Health: A Review. Crit. Rev. Food Sci. Nutr..

[21] Ovaskainen M.-L., Törrönen R., Koponen J.M., Sinkko H., Hellström J., Reinivuo H., Mattila P. (2008). Dietary intake and major food sources of polyphenols in Finnish adults. J. Nutr..

[22] Heim K.E., Tagliaferro A.R., Bobilya D.J. (2002). Flavonoid antioxidants: Chemistry, metabolism and structure-activity relationships. J. Nutr. Biochem..

[23] Møller C., Hansen S.H., Cornett C. (2009). Characterisation of tannin-containing herbal drugs by HPLC. Phytochem. Anal..

[24] Rasul A., Di J., Millimouno F., Malhi M., Tsuji I., Ali M., Li J., Li X. (2013). Reactive Oxygen Species Mediate Isoalantolactone-Induced Apoptosis in Human Prostate Cancer Cells. Molecules.

[25] Sarfraz I., Rasul A., Jabeen F., Younis T., Zahoor M.K., Arshad M., Ali M. (2017). Fraxinus: A Plant with Versatile Pharmacological and Biological Activities. Evid.-Based Complementary Altern. Med..

[26] Khanbabaee K., van Ree T. (2001). Tannins: Classification and definition. Nat. Prod. Rep..

[27] Jaiswal H., Singh O.J., Chauhan A., Sahu M.K., Dv S.P. (2018). A review on tannins. Biosci. J..

[28] Maji Amal K., Pratim B. (2017). Strychnos nux-vomica: A poisonous plant with various aspects of therapeutic significance. J. Basic Clin. Pharm..

[29] Duval A., Averous L. (2016). Characterization and physico-chemical properties of condensed tannins from Acacia catechu Characterization and physico-chemical properties of condensed tannins from Acacia catechu. J. Agric. Food Chem..

[30] Zengin G., Locatelli M., Carradori S., Mocan A.M., Aktumsek A. (2016). Total phenolics, flavonoids, condensed tannins content of eight centaurea species and their broad inhibitory activities against cholinesterase, tyrosinase, α-amylase and α-glucosidase. Not. Bot. Horti Agrobot. Cluj-Napoca.

[31] Calou I., Bandeira M.A., Aguiar-Galvão W., Cerqueira G., Siqueira R., Neves K.R., Brito G.A., Viana G. (2014). Neuroprotective Properties of a Standardized Extract from *Myracrodruon urundeuva* Fr. All. (Aroeira-Do-Sertão), as Evaluated by a Parkinson’s Disease Model in Rats. Parkinson’s Dis..

[32] Nile S.H., Park S.W. (2014). Edible berries: Bioactive components and their effect on human health. Nutrition.

[33] Afshari A.R., Sadeghnia H.R., Mollazadeh H. (2016). A Review on Potential Mechanisms of Terminalia chebula in Alzheimer’s Disease. Adv. Pharmacol. Sci..

[34] Kumar S., Brijeshlata D.S., Dixit S. (2012). Screening of traditional indian spices for inhibitory activity of acetylcholinesterase and butyrylcholinesterase enzymes. Int. J. Pharma Bio Sci..

[35] Dhivya P.S., Sobiya M., Selvamani P., Latha S. (2014). An approach to alzheimer’s disease treatment with cholinesterase inhibitory activity from various plant species. Int. J. Pharmtech Res..

[36] Olajide O.A., Kumar A., Velagapudi R., Okorji U.P., Fiebich B.L. (2014). Punicalagin inhibits neuroinflammation in LPS-activated rat primary microglia. Mol. Nutr. Food Res..

[37] Tapias V. (2014). Pomegranate juice exacerbates oxidative stress and nigrostriatal degeneration in Parkinson’s disease. Neurobiol. Aging.

[38] Kujawski R. (2016). Perspectives for gallotannins neuroprotective potential-current experimental evidences Comparison of extracts from root of rhodiola rosea inhibitory action on EtOH tolerance development in rats View project Małgorzata Kujawska. J. Med Sci..

[39] Thenmozhi A.J., Manivasagam T., Essa M.M. (2016). Role of plant polyphenols in Alzheimer’s disease. Adv. Neurobiol..

[40] Braidy N., Jugder B.-E., Poljak A., Jayasena T., Nabavi S.M., Sachdev P., Grant R. (2017). Molecular targets of tannic acid in Alzheimer’s disease. Curr. Alzheimer Res..

[41] Hung C.W., Chen Y.C., Hsieh W.L., Chiou S.H., Kao C.L. (2010). Ageing and neurodegenerative diseases. Ageing Res. Rev..

[42] Hussain G., Rasul A., Anwar H., Sohail M.U., Kamran S.K.S., Baig S.M., Shabbir A., Iqbal J. (2017). Epidemiological Data of Neurological Disorders in Pakistan and Neighboring Countries: A Review. Pak. J. Neurol. Sci..

[43] Kovacs G.G. (2014). Current Concepts of Neurodegenerative Diseases. EMJ Neurol.

[44] Association A. (2009). Alzheimer’s disease facts and figures. Alzheimer’s Dement..

[45] Cai Z., Wang C., Yang W. (2016). Role of berberine in Alzheimer’s disease. Neuropsychiatr. Dis. Treat..

[46] Prasanthi J.R.P., Dasari B., Marwarha G., Larson T., Chen X., Geiger J.D., Ghribi O. (2010). Caffeine protects against oxidative stress and Alzheimer’s disease-like pathology in rabbit hippocampus induced by cholesterol-enriched diet. Free Radic. Biol. Med..

[47] Zhu F., Wu F., Ma Y., Liu G., Li Z., Sun Y., Pei Z. (2011). Decrease in the production of beta-amyloid by berberine inhibition of the expression of beta-secretase in HEK293 cells. BMC Neurosci..

[48] Castellani R.J., Zhu X., Lee H., Moreira P.I., Perry G., Smith M.A. (2007). Neuropathology and treatment of Alzheimer disease: Did we lose the forest for the trees?. Expert Rev. Neurother..

[49] Heron M. (2012). Deaths: Leading causes for 2008. Natl. Vital Stat. Rep..

[50] Kumar A., Singh A. (2015). Pharmacological Reports Review article A review on Alzheimer’s disease pathophysiology and its management: An update. Pharm. Rep.

[51] Duffy F.H., Albert M.S., McAnulty G. (1984). Brain electrical activity in patients with presenile and senile dementia of the Alzheimer type. Ann. Neurol..

[52] Bhullar K.S., Rupasinghe H.P.V. (2013). Polyphenols: Multipotent Therapeutic Agents in Neurodegenerative Diseases. Oxidative Med. Cell. Longev..

[53] Van Bulck M., Sierra-Magro A., Alarcon-Gil J., Perez-Castillo A., Morales-Garcia J.A. (2019). Novel approaches for the treatment of Alzheimer’s and Parkinson’s disease. Int. J. Mol. Sci..

[54] Sarrafchi A., Bahmani M., Shirzad H., Rafieian-Kopaei M. (2016). Oxidative Stress and Parkinson’s Disease: New Hopes in Treatment with Herbal Antioxidants. Curr. Pharm. Des..

[55] Oguru M., Tachibana H., Toda K., Okuda B., Oka N. (2010). Apathy and depression in parkinson disease. J. Geriatr. Psychiatry Neurol..

[56] Neurol J.J. (2008). Parkinson’s disease: Clinical features and diagnosis. Psychiatry Interpers. Biol. Process..

[57] Gaki G.S., Papavassiliou A.G. (2014). Oxidative stress-induced signaling pathways implicated in the pathogenesis of Parkinson’s disease. Neuromol. Med..

[58] Magrinelli F., Picelli A., Tocco P., Federico A., Roncari L., Smania N., Zanette G., Tamburin S. (2016). Pathophysiology of Motor Dysfunction in Parkinson’s Disease as the Rationale for Drug Treatment and Rehabilitation. Parkinson’s Dis..

[59] Hussain G., Wang J., Rasul A., Anwar H., Imran A., Qasim M., Zafar S., Kamran S.K.S., Razzaq A., Aziz N. (2019). Role of cholesterol and sphingolipids in brain development and neurological diseases. Lipids Health Dis..

[60] Wideman T.H., Zautra A.J., Edwards R.R. (2014). Rethinking the fear avoidance model: Toward a multidimensional framework of pain-related disability. NIH Public Access..

[61] Hurley L.L., Tizabi Y. (2013). Neuroinflammation, neurodegeneration, and depression. Neurotox. Res..

[62] Bajpai A., Verma A.K., Srivastava M., Srivastava R. (2014). Oxidative stress and major depression. J. Clin. Diagn. Res..

[63] Cheng H.A., Drinnan C.T., Pleshko N., Fisher O.Z. (2015). Pseudotannins self-assembled into antioxidant complexes. Soft Matter.

[64] Wu X., Gu L., Prior R.L., McKay S., Nakajima J., Tanaka I., Seo S., Yamazaki M., Saito K. (2004). Characterization of anthocyanins and proanthocyanidins in some cultivars of Ribes, Aronia, and Sambucus and their antioxidant capacity. J. Agric. Food Chem..

[65] Bagchi D., Bagchi M., Stohs S.J., Das D.K., Ray S.D., Kuszynski C.A., Joshi S.S., Pruess H.G. (2000). Free radicals and grape seed proanthocyanidin extract: Importance in human health and disease prevention. Toxicology.

[66] Liu X., Li X.N., Bao L.X., Ling B.Y. (2005). Effect of grape seed extract proanthocyanidin on loaded swimming time in mice. Chin. J. Clin. Rehabil..

[67] Amarowicz R., Janiak M. (2018). Hydrolysable Tannins. Encyclopedia of Food Chemistry.

[68] Zhao J., Khan I.A., Fronczek F.R. (2011). Gallic acid. Acta Crystallogr. Sect. E Struct. Rep. Online.

[69] Daglia M., Di Lorenzo A., Nabavi S.F., Talas Z.S., Nabavi S.M. (2014). Polyphenols: Well beyond the Antioxidant Capacity: Gallic Acid and Related Compounds as Neuroprotective Agents: You are What You Eat!. Curr. Pharm. Biotechnol..

[70] Thompson M.A., Collins P.B. (2013). The anti-oxidative and anti-inflammatory roles of gallic acid on transcriptional regulation. Handbook on Gallic Acid.

[71] Dumitriu A., Moser C., Hadzi T.C., Williamson S.L., Pacheco C.D., Hendricks A.E., Latourelle J.C., Wilk J.B., Destefano A.L., Myers R.H. (2012). Postmortem Interval Influences α-Synuclein Expression in Parkinson Disease Brain. Parkinsons Dis..

[72] Sameri M.J., Sarkaki A., Farbood Y., Mansouri S.M.T. (2011). Motor disorders and impaired electrical power of pallidal EEG improved by gallic acid in animal model of Parkinson’s disease. Pak. J. Biol. Sci..

[73] Stoner G.D., Seeram N.P. (2011). Berries and Cancer Prevention.

[74] Jourdes M. (2013). Hydrolyzable tannins: Gallotannins and ellagitannins. Natural Products: Phytochemistry, Botany and Metabolism of Alkaloids, Phenolics and Terpenes.

[75] Reddy D.B. (2009). Reddy Chebulagic acid, a COX-LOX dual inhibitor isolated from the fruits of Terminalia chebula Retz., induces apoptosis in COLO-205 cell line. J. Ethnopharmacol..

[76] Park J.H., Joo H.S., Yoo K.Y., Shin B.N., Kim I.H., Lee C.H., Choi J.H., Byun K., Lee B., Lim S.S. (2011). Extract from Terminalia chebula seeds protect against experimental ischemic neuronal damage via maintaining SODs and BDNF levels. Neurochem. Res..

[77] Subash S., Essa M.M., Al-Adawi S., Memon M.A., Manivasagam T., Akbar M. (2014). Neuroprotective effects of berry fruits on neurodegenerative diseases. Neural Regen. Res..

[78] Seeram N.P., Momin R.A., Nair M.G., Bourquin L.D. (2001). Cyclooxygenase inhibitory and antioxidant cyanidin glycosides in cherries and berries. Phytomedicine.

[79] Ferencik M., Novak M., Rovensky J., Rybar I. (2001). Alzheimer’s disease, inflammation and non-steroidal anti-inflammatory drugs. Bratisl. Lek. Listy.

[80] Sylla T., Pouységu L., Dacosta G., Deffieux D., Monti J.P., Quideau S. (2015). Gallotannins and Tannic Acid: First Chemical Syntheses and in Vitro Inhibitory Activity on Alzheimer’s Amyloid β-Peptide Aggregation. Angew. Chem. Int. Ed..

[81] Sinclair A.J., Begg D., Mathai M., Weisinger R.S. (2007). Omega 3 fatty acids and the brain: Review of studies in depression. Asia Pac. J. Clin. Nutr..

[82] Gold P.W., Machado-Vieira R., Pavlatou M.G. (2015). Clinical and Biochemical Manifestations of Depression: Relation to the Neurobiology of Stress. Neural Plast..

[83] Chandra Shekar D., Manohar V.R., Rao S.N. (2012). Antidepressant activity of aqueous extract of fruits of terminalia chebula in rats. Int. J. Pharm. Pharm. Sci..

[84] Chandrasekhar Y., Ramya E.M., Navya K., Phani Kumar G., Anilakumar K.R. (2017). Antidepressant like effects of hydrolysable tannins of Terminalia catappa leaf extract via modulation of hippocampal plasticity and regulation of monoamine neurotransmitters subjected to chronic mild stress (CMS). Biomed. Pharmacother..

[85] Pariante C.M., Benkert O., Szegedi A., Müller M.J. (2015). Antidepressant Drugs. International Encyclopedia of the Social & Behavioral Sciences.

[86] Cai L., Li R., Tang W.J., Meng G., Hu X.Y., Wu T.N. (2015). Antidepressant-like effect of geniposide on chronic unpredictable mild stress-induced depressive rats by regulating the hypothalamus-pituitary-adrenal axis. Eur. Neuropsychopharmacol..

[87] Lin P., Campbell D.G., Chaney E.F., Liu C.F., Heagerty P., Felker B.L., Hedrick S.C. (2005). The influence of patient preference on depression treatment in primary care. Ann. Behav. Med..

[88] Robles H. (2014). Tannic Acid. Encyclopedia of Toxicology.

[89] Serrano J., Puupponen-Pimiä R., Dauer A., Aura A.M., Saura-Calixto F. (2009). Tannins: Current knowledge of food sources, intake, bioavailability and biological effects. Mol. Nutr. Food Res..

